# Proteomic Profile of *Brucella abortus*-Infected Bovine Chorioallantoic Membrane Explants

**DOI:** 10.1371/journal.pone.0154209

**Published:** 2016-04-22

**Authors:** Juliana P. S. Mol, Simone F. Pires, Alexander D. Chapeaurouge, Jonas Perales, Renato L. Santos, Hélida M. Andrade, Andrey P. Lage

**Affiliations:** 1 Universidade Federal de Minas Gerais, Escola de Veterinária, Departamento de Medicina Veterinária Preventiva, Belo Horizonte, Minas Gerais, Brazil; 2 Universidade Federal de Minas Gerais, Instituto de Ciências Biológicas, Departamento de Parasitologia, Belo Horizonte, Minas Gerais, Brazil; 3 Fundação Oswaldo Cruz, Instituto Oswaldo Cruz, Laboratório de Toxinologia, Rio de Janeiro, Rio de Janeiro, Brazil; 4 Universidade Federal de Minas Gerais, Escola de Veterinária, Departamento de Clínica e Cirurgia Veterinárias, Minas Gerais, Brasil; Universidad de Costa Rica, COSTA RICA

## Abstract

*Brucella abortus* is the etiological agent of bovine brucellosis, a zoonotic disease that causes significant economic losses worldwide. The differential proteomic profile of bovine chorioallantoic membrane (CAM) explants at early stages of infection with *B*. *abortus* (0.5, 2, 4, and 8 h) was determined. Analysis of CAM explants at 0.5 and 4 h showed the highest differences between uninfected and infected CAM explants, and therefore were used for the Differential Gel Electrophoresis (DIGE). A total of 103 spots were present in only one experimental group and were selected for identification by mass spectrometry (MALDI/ToF-ToF). Proteins only identified in extracts of CAM explants infected with *B*. *abortus* were related to recognition of PAMPs by TLR, production of reactive oxygen species, intracellular trafficking, and inflammation.

## Introduction

Brucellosis is considered one of the most important zoonosis. It is responsible for important economic losses due to abortion and culling of infected animals. In man, it causes a systemic febrile illness with a wide spectrum of symptoms, although arthritis is among the most frequent manifestations [1]. In cattle the main causative agent of brucellosis is *Brucella abortus* and during pregnancy it can infect and multiply intensely within trophoblastic cells of the placenta at late gestation causing necrotizing placentitis associated with abundant neutrophilic infiltrate [2].

The capacity of *B*. *abortus* to cause disease is related to its ability to invade host cells, survive intracellularly, and evade antimicrobial defenses of the host. However, the mechanisms related to this ability are not yet fully understood. It is known, however, that once *Brucella* spp. reaches the intracellular environment, the pathogen actively interferes with the host cell metabolism and defense favoring its survival and intracellular multiplication. *Brucella* spp. modulate intracellular trafficking by preventing maturation of phagosomes and blocking endosome-lysosome fusion, which prevents the degradation of bacteria [3,4].

Although *Brucella* spp. do not have classical virulence factors, these organisms have several known mechanisms associated with pathogenicity [4]. One of these mechanisms is associated with LPS, which differs from other Gram-negative bacteria. *Brucella* spp. LPS is a poor inducer of oxidative burst, reactive nitrogen intermediates, and secretion of lysozyme [5]. Some studies have also shown that *Brucella* spp. LPS reduces TLR4 (Toll-like receptor 4) agonistic activity and that despite being recognized by the receptor, this interaction does not induce cytokine production [6]. The *virB* operon-encoded type IV secretion system (T4SS) is a key virulence factor for *Brucella* spp. This T4SS secretes effector proteins through the envelope of the bacterial cell, and it is required for intracellular survival and *in vivo* persistence of *Brucella* and this expression is induced by acidification of the phagosome after phagocytosis [7]. Effector proteins secreted through the T4SS modulates maturation of the *Brucella*-containing vacuole (BCV), preventing fusion of the BCV with lysosomes at early stages of infection, and driving the interaction between BCV and endoplasmic reticulum (ER) and subsequent maturation of BCV at later stages of infection [8,9]. Two *Brucella* proteins containing a TIR (Toll intracellular domain/interleukin-1) domain, namely BtpA and BtpB, have been identified in *Brucella* spp. BtpA and BtpB modulate the host inflammatory responses during *Brucella* sp. infection by interfering with TLR signaling [10–12].

Proteomic analyzes in the context of the interaction between *B*. *abortus* and its target cells are also scarce. These studies are very challenging due to the high complexity of samples and very low concentrations of certain proteins, requiring the use of highly sensitive analytical techniques. All proteomic studies reported to date have used the model of infection of phagocytic cells, whereas the profile of protein expression by trophoblastic cells infected with *B*. *abortus* have not been previously studied [13–18]. In this study, *ex vivo* culture of trophoblastic cells in CAM explants was associated with proteomic analysis to study the interaction between *B*. *abortus* and trophoblastic cells.

## Materials and Methods

### Bacterial strain and growth conditions

The inoculum was prepared from cultures of *B*. *abortus* 2308 grown in 20 mL of tryptic soy broth (Difco, USA) for 12–15 h at 37°C under agitation (200 rpm). After incubation, optical density of bacterial suspensions was determined by spectrophotometry (OD_600_) and adjusted to 1.0 x 10^8^ bacteria/mL. Number of bacterial cells was confirmed by serially diluting in PBS (pH 7.4), and plating 100 μL of each dilution on tryptic soy agar (Difco) in duplicate. After 48 h of incubation at 37°C with 5% CO_2_, colonies were counted and the number of colony forming units (CFU) was obtained by averaging the duplicates. CFU numbers were determined by the drop count method [19]. Manipulation of *B*. *abortus* was performed under biosafety level 3 conditions [20].

### Infection of chorioallantoic membrane (CAM) explants with *Brucella abortus* 2308

CAM explants were obtained from three intact bovine uteruses at the final third of gestation collected at a local slaughterhouse (Frigorífico Uberaba Ltda, Sabará, Minas Gerais), as previously described [21]. Gestational age was estimated by measuring the cephalococcygeal length (Crown-rump length) [22]. Only placentas from *Brucella*-free fetuses, based on rose Bengal plate agglutination test using amniotic fluid, were included in these experiments. This study was approved by the Committee for Ethical use of Experimental Animals of the Universidade Federal de Minas Gerais, Brazil (CETEA), under protocol 183/2010.

Chorioallantoic membranes were aseptically removed from the uterus and immediately placed into RPMI 1640 (Roswell Park Memorial Institute) medium (Invitrogen, USA) with 50 U/mL of penicillin and 50 g/mL of streptomycin (Invitrogen, USA) for 20 min and were then washed two times with RPMI 1640 to remove antibiotic residues. Explants were prepared using snapwell inserts (Snapwell^™^ Inserts—Corning, USA) and placed into six-well cell culture plates (Corning) with supplemented medium (RPMI 1640 with 4 mM glutamine, 1 mM pyruvate, 1 mM non-essential amino acids, 2.9 mM sodium bicarbonate, 15% fetal bovine serum) in contact with the trophoblastic and allantoic or amniotic surfaces. The central area of each explant was inoculated with 200 μL of culture medium (supplemented RPMI 1640) containing 2.0 x 10^7^ CFU, which correspond to a multiplicity of infection (MOI) of approximately 1000 [21]. The explants were centrifuged for 15 min at 1000 xg and maintained at 37°C in 5% CO_2_ for 30 min to allow internalization of bacteria. Extracellular bacteria were eliminated by adding 200 μL of medium with gentamicin (50 mg/mL) (Invitrogen), followed by incubation for 1 h at 37°C under 5% CO_2_. After incubation, medium containing gentamicin was removed, the explants were gently washed twice with PBS (phosphate buffered saline—pH 7.4) and then, 200 μL of supplemented RPMI 1640 medium was added in each explant. In this assay, each group was evaluated in triplicate at 0.5, 2, 4, and 8 h after the removal of the gentamicin supplemented RPMI 1640. Explants in the control group were inoculated with sterile supplemented RPMI 1640 medium and submitted to the same conditions.

### Internalization of *Brucella abortus* by trophoblastic cells of CAM explants

Three explants from each group (uninfected and infected) obtained from the chorioallantoic membrane of three fetuses were used to determine the number of internalized bacteria. After removal of the RPMI culture medium with gentamycin, the explants were washed twice in PBS pH 7.4 and then lysed with 200 μL of sterile 0.1% Triton X-100 (Roche, Germany). Lysates were serially diluted in PBS pH 7.4 and 100 μL of each dilution were plated on Tryptic Soy Agar (Difco, USA) in duplicate. After 48 h of incubation at 37°C in 5% CO_2_, colony counts were performed and the number of CFU was obtained from the average of triplicates. CFU numbers underwent logarithmic transformation followed by analysis of variance (ANOVA). Comparison of means was performed using the Tukey's multiple comparison test with significance level of P≤0.05 [23].

### Extraction of proteins from CAM explants

At 0.5, 2, 4, and 8 h after the removal of the medium containing gentamicin, CAM explants were washed three times with RPMI 1640 medium without bicarbonate and without fetal bovine serum. Protein extraction was performed using 200 μL of lysis buffer (8 M urea, 2 M thiourea, 4% w/v CHAPS, 40 mM Tris 1M) containing protease inhibitors (GE Healthcare, UK), which was added to the trophoblastic surface of CAM explants. The explants were maintained under agitation (50 rpm) for 30 min on an orbital shaker (IKA Labortechnik, Germany). Lysates were centrifuged for 30 min at 20.000 xg at room temperature. The supernatant was collected and kept at -80°C. Protein concentration was measured using the 2D Quant Kit (GE Healthcare, UK).

### Two-dimensional gel electrophoresis (2DE)

In order to evaluate reproducibility and to determine the time points to be evaluated by DIGE, the proteomic profile was assessed initially using 7 cm gels, pH 4–7. Extracted proteins (50 μg) from CAM explants were added to the rehydration solution (1.25 μL IPG buffer, pH 4–7, 10 μL/L—GE Healthcare, UK) and IEF buffer (8 M urea, 2 M thiourea, 4% CHAPS, 0.0025% bromophenol blue, 10 mg/mL dithiothreitol—GE Healthcare) in a total volume of 125 μL per IPG strip (7 cm, pH 4–7) (GE Healthcare). Samples were incubated with IPG strips on rehydration apparatus (Immobiline DryStrip Reswelling Tray, GE Healthcare, UK) for 12 h and subjected to isoelectric focusing using the Ettan^™^ IPGphor^™^ 3 Isoelectric Focusing System (GE Healthcare, UK) and the program IPGphor (GE Healthcare, UK). Focused IPG strips were incubated for 15 min in equilibration solution (50 mM Tris-HCl, pH 8.8, 6 M urea, 30% glycerol, 2% SDS, 0.002% bromophenol blue, and 125 mM DTT) and then alkylated for further 15 min in an equilibration solution containing 13.5 mM iodocetamide (GE Healthcare, UK) instead of DTT. Electrophoresis was performed in 12% polyacrylamide gel containing SDS in a vertical electrophoresis apparatus (Ettan DALTsix electrophoresis Unit -GE Healthcare, UK) at 30 mA per gel. Bench Mark Protein^®^ Lader (Invitrogen, USA) was used as molecular mass marker. Gels were stained with Colloidal Coomassie Brilliant blue G-250 (GE Healthcare, UK) [24] and digitized using the Image Scanner (Amersham Biosciences, England).

### Differential gel electrophoresis (DIGE)

Protein samples extracted from CAM explants were labeled using the CyDye DIGE Fluors (minimal dyes) for Ettan DIGE kit (GE Healthcare, UK) according to the manufacturer. To a pool containing 50 μg of an equal mixture of proteins extracted from infected and uninfected CAM explants produced from three fetuses, 400 pmol of dyes were added. Dye swap was performed and Cy2 dye was used as internal standard. Labeled samples and 800 μg of mixture of unlabeled proteins were incubated with the IPG strip (18 cm, pH 4–7—GE Healthcare) in a rehydration apparatus (Immobiline DryStrip Reswelling Tray, GE Healthcare, UK) for approximately 12 h and subjected to isoelectric focus using Ettan^™^ IPGphor^™^ 3 Isoelectric Focusing System (GE Healthcare, UK) and the program IPGphor (GE Healthcare, UK). Electrophoresis was performed in 12% polyacrylamide gel containing SDS in vertical electrophoresis apparatus (Ettan DALTsix Electrophoresis Unit—GE Healthcare, UK) and scanned using Typhoon Trio (GE Healthcare, UK) with excitation/emission wave lengths specific for Cy2 (488/520 nm), Cy3 (532/580 nm), and Cy5 (633/670 nm). After scanning, gels were stained with Coomassie Brilliant blue G-250 (Thermo Scientific, USA) for marking the spots of interest.

### Image analysis of the gels

The 2D Image Master Platinum^™^ software (version 6.0, GE Healthcare, UK) was used for image analysis of the two-dimensional gels (2DE) stained with Coomassie Brilliant blue G-250 by a combination of automatic detection and manual detection of spots. To determine the relative amount of each spot was used the method of normalization volume. Mean volumes of each spot were calculated by the software, and spots with at least two-fold increase or decrease were considered for further analysis. Means were compared by the Student t test and considered significant when P≤0.05 [23]. For the fluorescent gels, DeCyder^™^ 2-D Differential Analysis v7.0 software (GE Healthcare, UK) was used according to the manufacturer's instructions. The spots containing proteins labeled with different fluorescent dyes were co-detected and quantified in the three images obtained from each gel. Based on the ratio of the average volume normalized spots that had at least a two-fold increase or decrease was evaluated for statistical significance. Data was subjected to analysis of variance (ANOVA) and means compared using the Student t test with significance level of P≤0.05 [23]. Spots with significant changes were selected for identification by mass spectrometry.

### In gel proteolysis and mass spectrometry (MS)

Selected spots were cut in pieces of approximately 1.0 mm^3^ using sterile scalpel. Discoloration of the gel with 400 μL of a solution containing 25 mM ammonium bicarbonate (NH_4_CO_3_) pH 8.0 (Synth, Brasil) and 50% acetonitrile (Sigma-Aldrich, USA) was repeated three times for 15 min at room temperature under vigorous agitation. Gel fragments were dehydrated with 200 μL of acetonitrile for 5 min and dried under vacuum centrifugation. Proteins were digested using 20 μL of a solution containing 2.0 mM of NH_4_CO_3_ (Synth, Brasil) and 20 ng/μL trypsin (Promega, USA), followed by incubation for 16–24 h at 37°C. Peptides were extracted with 5% formic acid (Sigma-Aldrich, USA) and 50% acetonitrile. Protein extracts were concentrated (final volume of 10 μL) in ZipTip^®^ C^18^columns (Millipore, USA) and the final volume was reduced to 5.0 μL in a vacuum centrifuge. For sample analysis in the mass spectrometer, 0.3 μL of sample solution was mixed with the same volume of saturated matrix solution [10 mg/mL R-cyano-4-hydroxycinnamic acid in 50% acetonitrile/0.1% trifluoroacetic acid] (Sigma-Aldrich, USA). Raw data for identifying proteins were obtained from ABSCIEX Proteomics Analyzer MALDITOF/TOF^™^ System 5800 (Applied Biosystems, USA). The external calibration mode MS was performed using a mixture of four peptides: des-arg1-bradykinin (m/z = 904,468), angiotensin I (m/z = 1296.685); Glu1-fibrinopeptide B (m/z = 1570.677) and adrenocorticotropic hormone (18–39) (m/z = 2465.199) (Applied Biosystems, USA). The MS/MS spectra were calibrated externally using known masses of fragmentations observed in the MS/MS angiotensin I. After data acquisition, a list of peaks was obtained from the raw data of the MS/MS using the Mascot to function Peaks 4000 Series Explore software (Applied Biosystems, USA).

### Identification of proteins in the database

All information acquired for each spot, i.e. the mass/charge ratio and intensity of peaks, obtained in the spectrum and the reasons mass/charge and intensities of the peaks relating to each of the five spectra MS/MS has been compiled into one text file. This file was used by MASCOT (Matrix Science, USA) (http://www.matrixscience.com) to perform the search in databases. Once complete the search, MASCOT reports the results in a bar graph in which there is a value taken as the limit. Scores below this value indicates random events, i.e. no statistical value. On the other hand, if the score assigned to a given protein exceeds the threshold value identifying the chance of an event being generated randomly is 5% and the larger this value the greater the probability of being correct.

The parameters used in the search were: no restriction for protein molecular weight, a trypsin cleavage site lost, variable modifications of methionine (oxidation) and cysteine (carbamidomethylation), formation of pyroglutamate at the N-terminal glutamine without other post-translational modifications. The mass tolerance for peptides in searches was 0.8 Da for MS spectra and 0.6 Da for MS/MS spectra. The data bases of *Bos taurus* and *Brucella abortus* available at NCBI (National Center for Biotechnology Information, USA) were used.

## Results

### Internalization of *Brucella abortus* 2308 in bovine trophoblastic cells of CAM explants

**Fig 1:**
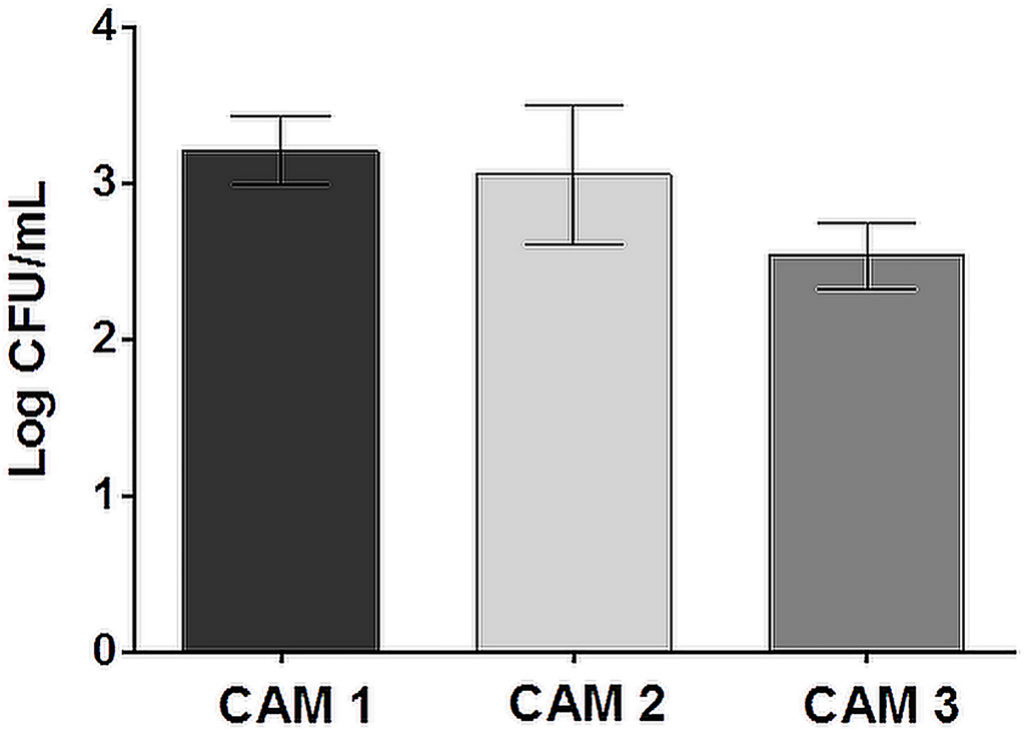
Number of *Brucella abortus* internalized (Log CFU/mL) in bovine CAM explants. Chorioallantoic membrane (CAM) explants were inoculated with *Brucella abortus* 2308, centrifuged for 15 min at 1000 xg and maintained at 37°C in 5% CO_2_ for 30 min to allow internalization of bacteria, followed by 1 h of incubation with gentamicin, and then lysed for intracellular CFU counting. Quantification of *B*. *abortus* internalized was performed using the drop count method [19] and data represent mean and standard error of three explants for each fetuses (n = 3), after logarithmic transformation. Analysis of variance (ANOVA) and Tukey's multiple comparison test were performed and no statistically significant differences were observed (P > 0.05).

Considering that placentitis and abortion are the most important manifestation of *B*. *abortus* infection in cattle [2], and that those are key steps for transmission of the disease, in this study we used a previously developed bovine CAM explant model of infection [21] to evaluate differentially expressed host proteins during the early stages of infection of trophoblastic cells with *B*. *abortus*. In order to ensure that the three independent experiments were in fact comparable, the number of bacteria located within trophoblastic cells, as previously demonstrated [21], was determined after inoculation of CAM explants with *Brucella abortus* 2308, centrifugation for 15 min at 1000 xg and incubation at 37°C in 5% CO_2_ for 30 min, followed by 1 h of incubation with gentamicin. The Fig 1 shows the average number of internalized bacteria (Log CFU/mL) in three explants (triplicates) prepared from three independent experiments (different fetuses). There were no significant differences in the number of *Brucella abortus* internalized in CAM explants from different fetuses under these conditions.

### Protein expression profile of CAM explants infected with *Brucella abortus*

The 2D gels (7 cm, pH 4–7) were compared to verify the reproducibility and the occurrence of experimental variation related to the quantity of protein extract added to the gel or/and intensity of the dye. According previous study, 2D gels are considered reproducible when presenting percentage of matching spots higher than 80%, and correlation ratio higher than 0.75 [25]. We observed that at each time point after infection, 2D gels had high reproducibility when the triplicates of uninfected CAM explants (% matching spots> 84%; correlation ration > 0.77) and triplicate of infected CAM explants (% matching spots> 88%; correlation ratio > 0.80) were compared. The proteomic profile of uninfected CAM explants remained stable throughout the course of the experiments (% matching spots = 77%). The same was observed in the profile of infected CAM explants (% matching spots = 80%). No significant changes were observed when the proteomic profile of uninfected CAM explants at various time points were compared. In contrast, four spots with significant changes in volume over time were detected in gels of infected explants: one of the spots was significantly increased at 2 h when compared to 4h, and the other three spots were significantly increased at 4 h when compared to 8 h (S1 Fig).

To determine the times points to be used for DIGE, an inter-class analysis in which the gels of uninfected and infected samples were compared in the same time post infection, was performed. There were no significant differences in volume of spots when infected and uninfected explants were compared, in each times evaluated. However, spots present in a single experimental group were detected (qualitative differences). Thus, 0.5 and 4 h post infection were chosen to be evaluated by the DIGE since these time points had the highest qualitative differences (S1 Table).

### Differential gel electrophoresis (DIGE)

**Fig 2:**
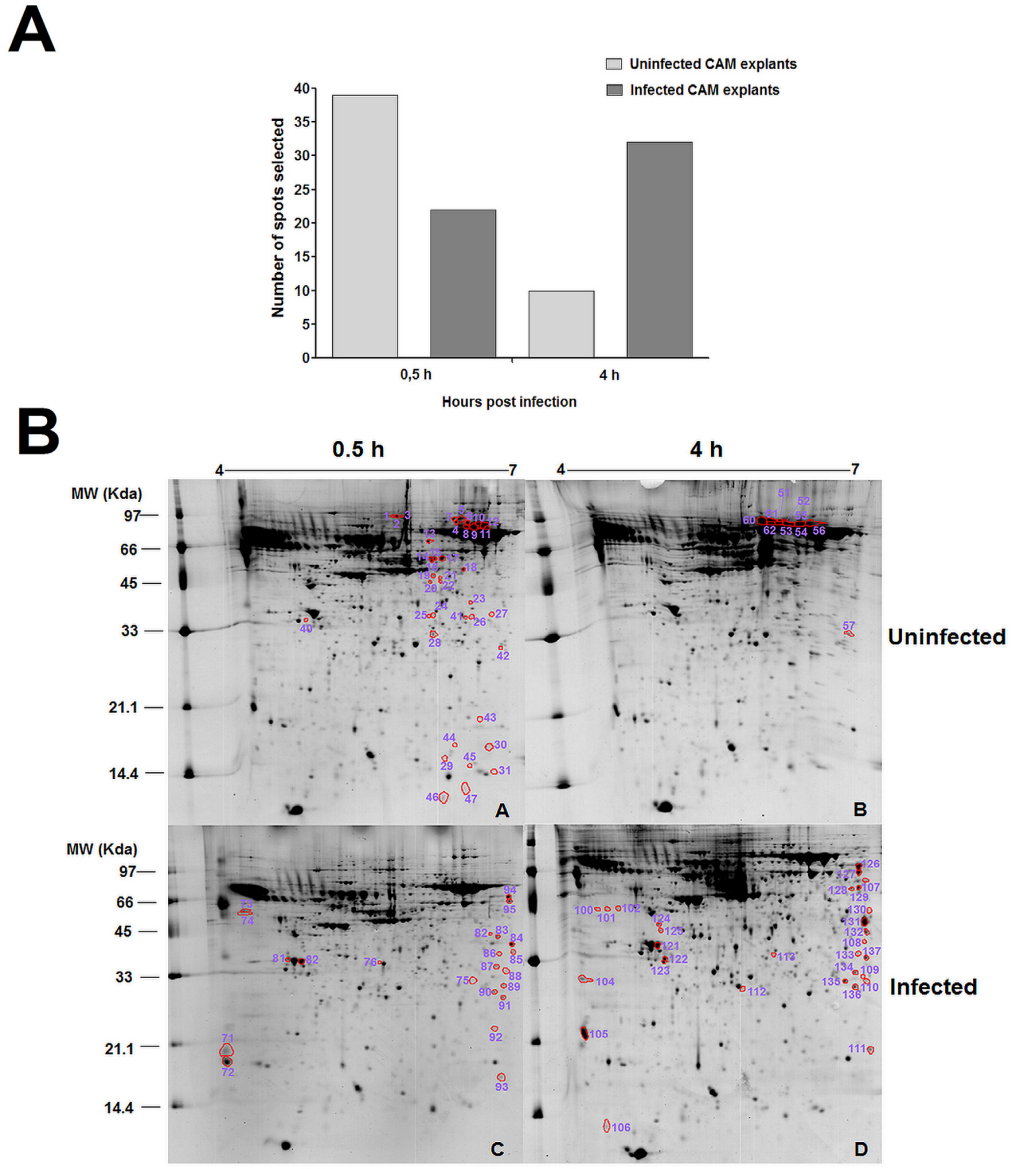
Two-dimensional gels stained with Coomassie Blue with samples from bovine CAM explants uninfected or infected with *B*. *abortus* at 0.5 or 4 h post infection. A. Number of spots selected for identification by mass spectrometry after DIGE analysis in infected and uninfected CAM explants at different time intervals after *B*. *abortus* 2308 infection. Differentially expressed spots were selected (i.e. spots that were present in one experimental group—infected or uninfected controls—and absent in the other). B. After analysis of DIGE gels using DeCyder^™^2-D Differential Analysis v7.0 software (GE Healthcare, UK) for determination of protein expression levels, new two-dimensional gels were prepared, stained with Coomassie Brilliant Blue G-250, and scanned for selecting spots of interest. Numbers refer to the spot identification used in the S1 Table.

In the analysis by DIGE, there were no statistically significant differences in the volume of the spots when gels from infected or uninfected explants were compared, considering significant in this analysis, values of fold change > 2 and P≥0.05. Interestingly, although no significant differences were observed in the volume of spots detected in both experimental groups at both time points, several spots were detected in only one experimental group (qualitative differences), as well was observed in 2D gels analysis. These spots were selected and numbered for identification by mass spectrometry (MALDI TOF/TOF) (Fig 2).

**Fig 3:**
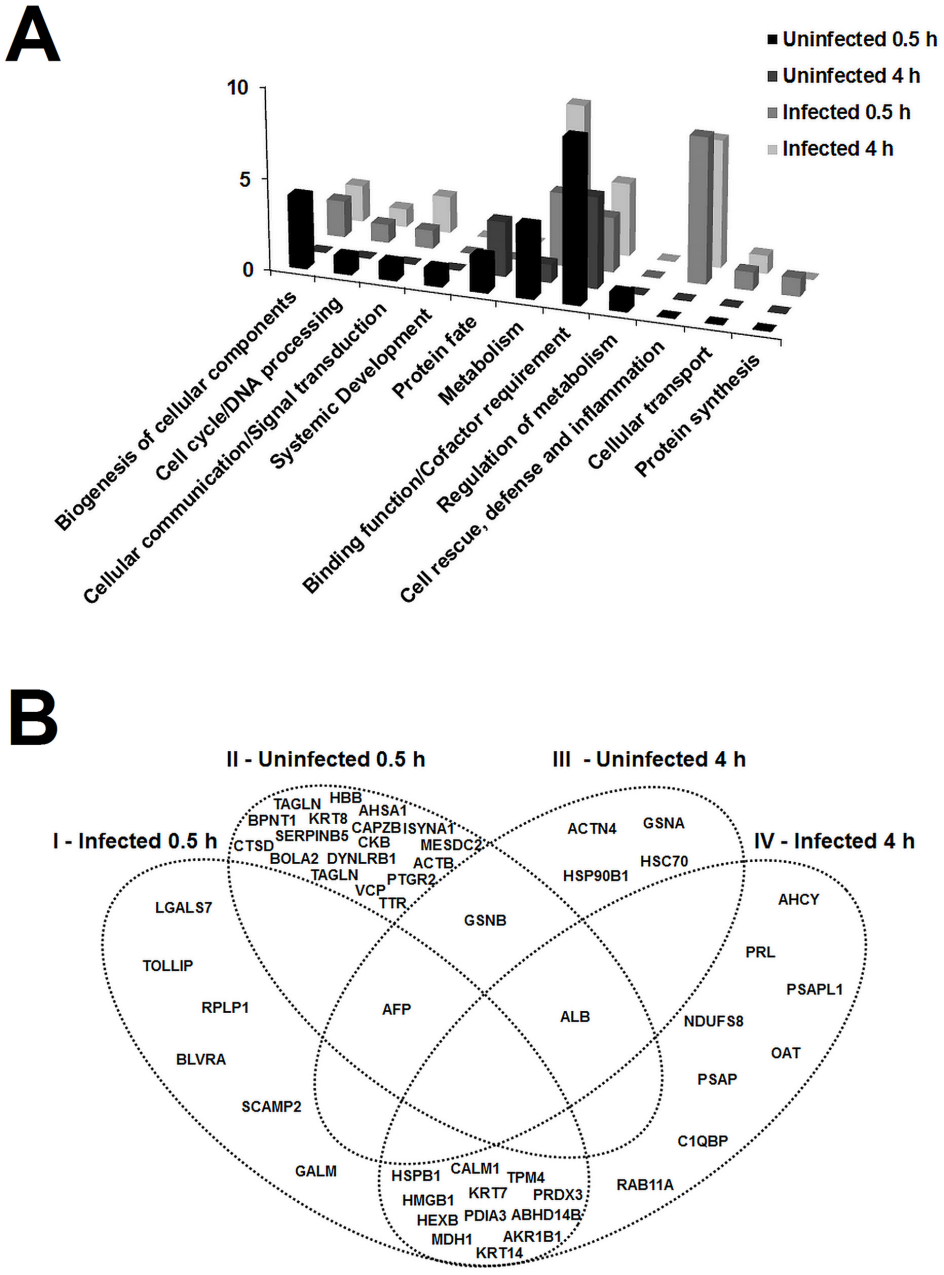
Functional classification and Venn diagram of differentially expressed proteins identified by mass spectrometry (MALDI TOF/TOF). A. Distribution of functional classification of proteins selected by DIGE analysis and identified by mass spectrometry in extracts of bovine CAM explants uninfected or infected with *Brucella abortus* at 0.5 h and 4 h post inoculation. B. Venn diagram showing differentially expressed proteins in each experimental group. Abbreviations: 3'(2'),5'-bisphosphate nucleotidase 1 (BPNT1), 3-hydroxyisobutyrate dehydrogenase, mitochondrial precursor (HIBADH), aldose 1-epimerase (GALM); abhydrolase domain-containing protein 14B (ABHD14B), activator of 90 kDa heat shock protein ATPase homolog 1 (AHSA1), adenosylhomocysteinase (AHCY), aldose reductase (AKR1B1), alpha-actinin-4 (ACTN4), alpha-fetoprotein precursor (AFP), beta-actin (ACTB), beta-hexosaminidase subunit beta preproprotein (HEXB), biliverdin reductase A (BLVRA), calmodulin (CALM1), cathepsin D (CTSD), complement component 1 Q subcomponent-binding protein, mitochondrial precursor (C1QBP), creatine kinase B-type (CKB), cytokeratin 8 (KRT8), dynein light chain roadblock-type 1 (DYNLRB1), endoplasmin precursor (HSP90B1), F-actin-capping protein subunit beta (CAPZB), galectin-7-like (LGALS7), gelsolin isoform a (GSNA), gelsolin isoform b (GSNB) heat shock cognate 71 kDa protein (HSC70), heat shock protein beta-1 (HSPB1), hemoglobin subunit beta (HBB), high-mobility group box 1-like (HMGB1), inositol-3-phosphate synthase 1 (ISYNA1), keratin 14-like (KRT14), keratin, type II cytoskeletal 7 (KRT7), LDLR chaperone MESD (MESDC2), malate dehydrogenase, cytoplasmic (MDH1), NADH dehydrogenase [ubiquinone] iron-sulfur protein 8 (NDUFS8), ornithine aminotransferase, mitochondrial precursor (OAT), placental prolactin (PRCII), proactivator polypeptide/prosaposin (PSAPL1/PSAP), protein disulfide-isomerase A3 precursor (PDIA3), prostaglandin reductase 2 (PTGR2), ras-related protein Rab-11A (RAB11A), ribosomal protein P1-like isoform 1 (RPLP1), secretory carrier-associated membrane protein 2 (SCAMP2), serine (or cysteine) proteinase inhibitor, clade B (ovalbumin), member 5 (**S**ERPINB5), similar to BolA-like protein 2 isoform 1 (BOLA2), thioredoxin-dependent peroxide reductase, mitochondrial precursor (PRDX3), toll-interacting protein (TOLLIP), transgelin-2 (TAGLN), transitional endoplasmic reticulum ATPase (VCP), transthyretin precursor (TTR), tropomyosin 4 (TPM4).

From a total of 103 spots analyzed by mass spectrometry, 74 (72%) were identified, from which 73 (98.64%) corresponded to proteins produced by the host (*Bos taurus*) (S2 Table). Only peptides from the spot number 129, identified as adenosylhomocysteinase, matched with significant scores *B*. *taurus* and *B*. *abortus* (S2 Table). Importantly, in this case, search in *Bos taurus* database resulted in identification of seven peptides with high value score (378–17% coverage), while search of the *B*. *abortus* data base identified one peptide with a low score (61–4% coverage), which indicates a greater probability that this protein was in fact expressed by bovine cells. The 74 spots identified corresponded to 51 proteins, i.e. the same protein was identified in different spots. All of them were classified into 11 functional categories by Funcat (Functional Classification of Proteins) (MIPS—Munich Information Center for Protein) (Fig 3A and Table 1). In order to better understand the distribution of proteins according to their function and experimental group from which they were identified, a Venn diagram showing the identified proteins in uninfected and infected CAM explants at 0.5 and 4 h was constructed (Fig 3B).

**Table 1:** Functional classification, subcellular localization and the experimental group which were identified the proteins expressed in uninfected CAM explants or CAM explants infected with *Brucella abortus* 2308 at 0.5 and 4 h post inoculation.

Spots*	Functional Classification of Proteins	Subcellular localization	Total
	**Biogenesis of cellular components**		6
14, 15, 16 and 17	cytokeratin 8 (370 AA)	Plasma membrane/Intermediate filaments	
25	F-actin-capping protein subunit beta	Cytoplasm/Actin cytoskeleton	
29	transgelin-2	Cytoplasm	
31	dynein light chain roadblock-type 1	Cytoskeleton/Cytoplasm	
74 and 130	keratin 14-like, partial	Intermediate filaments	
95 and 127	keratin, type II cytoskeletal 7	Intermediate filaments	
	**Cell cycle/ DNA processing**		
45	similar to BolA-like protein 2 isoform 1	Secreted	2
71, 72 and 105	Calmodulin	Cytoplasm/Actin cytoskeleton	
	**Cell rescue, defense and inflammation**		10
76	toll-interacting protein	Cytoplasm	
82	biliverdin reductase A	Cytoplasm	
83	aldose 1-epimerase	Cytoplasm	
84 and 131	aldose reductase	Cytoplasm	
88 and 137	high-mobility group box 1-like	Cytoplasm/Nucleus/Extracellular protein	
89 and 134	heat shock protein beta-1	Plasma membrane/Cytoplasm /Nucleus	
90 and 135	thioredoxin-dependent peroxide reductase, mitochondrial precursor	Mitochondrion	
94 and 126	Protein disulfide-isomerase A3 precursor	Endoplasmic reticulum	
103	complement component 1 Q subcomponent-binding protein, mitochondrial precursor	Mitochondrion/Mitochondrial Matrix	
112	NADH dehydrogenase [ubiquinone] iron-sulfur protein 8, mitochondrial precursor	Mitochondrion	
	**Cellular communication/signal transduction**		1
100, 101 and 102	placental prolactin related protein 2 precursor (PRL)	Nucleus/extracellular matrix component	
	**Cellular transport**		2
80	secretory carrier-associated membrane protein 2	Golgi Apparatus/trans-Golgi network membrane	
104	ras-related protein Rab-11A	Golgi Apparatus/vesicles/endossome	
	**Systemic Development**		1
43	LDLR chaperone MESD	Endoplasmic reticulum	
	**Metabolism**		11
13	inositol-3-phosphate synthase 1	Cytoplasm	
18	creatine kinase B-type	Cytoplasm/Mitochondrion	
21 and 22	3'(2'),5'-bisphosphate nucleotidase 1	Cytoplasm	
30	transthyretin precursor	Secreted	
84 and 131	malate dehydrogenase, cytoplasmic	Cytoplasm	
91 and 136	abhydrolase domain-containing protein 14B	Cytoplasm/Nucleus	
95 and 127	beta-hexosaminidase subunit beta preproprotein	Vacuole or lysosome	
106	proactivator polypeptide	Vacuole or lysosome	
106	Prosaposin	Mitochondrion/Vacuole or lysosome	
128	ornithine aminotransferase, mitochondrial precursor	Mitochondrion	
129	Adenosylhomocysteinase	Cytoplasm	
	**Protein fate**		4
18	cathepsin D	Mitochondrion/Vacuole or lysosome	
19	activator of 90 kDa heat shock protein ATPase homolog 1	Cytoplasm/Endoplasmic reticulum	
51, 60 and 61	endoplasmin precursor	Endoplasmic reticulum	
62	heat shock cognate 71 kDa protein	Cytoplasm	
	**Protein synthesis**		1
71	ribosomal protein P1-like isoform 1	Cytoplasm	
	**Binding function/Cofactor requirement**		12
1 and 2	transitional endoplasmic reticulum ATPase	Cytoplasm/Endoplasmic reticulum	
3, 5 and 55	gelsolin isoform b	Cytoskeleton	
6, 7, 12, 53, 54, 55 and 86	alpha-fetoprotein precursor	Cytoplasm/Extracellular/Secreted	
8, 9, 10, 11, 56, 107	albumin protein	Extracellular matrix component/Cytoplasm	
18	beta actin	Cytoplasm/Cytoskeleton	
20	prostaglandin reductase 2	Cytoplasm	
26	3-hydroxyisobutyrate dehydrogenase, mitochondrial precursor	Mitochondrion	
29	hemoglobin subunit beta	Mitochondrion	
51	alpha-actinin-4	Cytoplasm/Actin cytoskeleton	
53, 54 and 55	gelsolin isoform a	Cytoskeleton	
80, 81, 121 and 124	tropomyosin 4 isoform 2	Cytoplasm/Cytoskeleton	
93	galectin-7-like	Cytoplasm	
	**Regulation of metabolism**		
19	serine (or cysteine) proteinase inhibitor, clade B (ovalbumin), member 5	Secreted/Extracellular space	1
Total			51

In uninfected CAM explants group (0.5 and 4 h) were mainly identified proteins related to the biogenesis of cellular components (cytokeratin 8, dynein light chain roadblock-type 1, F-actin-capping protein subunit beta, transgelin), proteins with binding or cofactor function (3-hydroxyisobutyrate dehydrogenase, mitochondrial precursor, albumin protein, alpha-actinin-4, alpha-fetoprotein precursor, beta actin, gelsolin isoform a, gelsolin isoform b, hemoglobin subunit beta, prostaglandin reductase 2, transitional endoplasmic reticulum ATPase) but also related to the cell structure (gelsolin isoform a, gelsolin isoform b, alpha-actinin-4), proteins related to the metabolism (3'(2'),5'-nucleotidase bisphosphate 1 3-hydroxyisobutirate dehydrogenase, creatine kinase B-type, inositol-3-phosphate synthase 1, precursor transthyretin) and related to protein fate (activator of 90 kDa heat shock protein ATPase homolog 1, cathepsin D, endoplasmin precursor, heat shock cognate 71 kDa protein) (Fig 3A and 3B). It is also important to emphasize that most of the spots in which these proteins were identified, were selected based on the comparison between the two groups of uninfected CAM explants (77,5%, 38/49). Together these findings suggest that the differentially expressed proteins observed in uninfected CAM explants are related to the maintenance and stabilization of cellular organization and adaptation to cell culture after a stress condition related to the process of CAM explants preparation.

In addition, in *B*. *abortus*-infected CAM explants group the highest number of proteins identified was classified as related to metabolism or cell rescue defense and inflammation. Proteins with cell rescue, defense and inflammation functions (n = 15) were identified only in explants infected with *B*. *abortus* when compared to uninfected explants: 8 (53,3%) proteins identified 0,5 h post infection (aldose 1-epimerase, aldose reductase, biliverdin reductase A, heat shock protein beta-1, high-mobility group box 1, protein disulfide-isomerase A3 precursor, thioredoxin-dependent peroxide reductase and toll-interacting protein) and 7 (46,7%) proteins identified 4 h post infection (aldose reductase, complement component 1 Q subcomponent-binding protein, heat shock protein beta-1, high-mobility group box 1, NADH dehydrogenase [ubiquinone] iron-sulfur protein 8, protein disulfide-isomerase A3 precursor and thioredoxin-dependent peroxide reductase). Moreover, the proteins aldose 1-epimerase, heat shock protein beta-1, high-mobility group box 1-like protein disulfide-isomerase A3 precursor thioredoxin-dependent reductase peroxide, mitochondrial precursor, has been identified in both groups of infected explants (Fig 3A and 3B). The proteins albumin, alpha-fetoprotein precursor and gelsolin isoform b were identified in more than one experimental group but with different values of pI or molecular mass indicating possible post-translational modifications (Fig 3B and S3 Table).

## Discussion

Despite being bovine brucellosis a zoonotic disease that causes significant economic losses worldwide due to abortion and culling of infected animals, the pathogenic mechanisms are not yet fully understood.

Considering our results with what has been reported in the literature, we suggest the hypothesis that at earlier stages of infection with virulent *B*. *abortus* 2308, bovine CAM explants exhibited increase abundance of proteins directed to recognition of bacteria, mostly activation of the innate immune response, involving proteins related to TLR signaling and ROS production, as well as expression of proteins associated with intracellular trafficking and inflammation. Accordingly, we will direct our discussion to the simultaneous involvement of the proteins identified here and that have been previously linked to *Brucella* and their interactions.

One of the most interesting proteins identified in CAM explants during the initial stages of infection with *B*. *abortus* was the Toll-interacting protein (TOLLIP), a protein that modulates TLR signaling and also control membrane trafficking processes by its interaction with proteins and phosphoinositides [26]. As TOLLIP is a protein that negatively modulates the inflammatory response, expression of this protein in CAM explants infected by *B*. *abortus* at 0.5 h post inoculation may be related to the state of immunosuppression observed at early stages of infection [21], condition that can contribute to bacteria evasion of initial immune response, important step in the establishment of infection. The suppression of proinflammatory response by trophoblastic cells at early stages of *B*. *abortus* infection has been previously shown since transcriptomic analysis demonstrated a reduction of transcription of genes associated with TNF superfamily, e.g. lymphotoxin beta, tumor necrosis factor, and ligand chemokine—CXC motif at 4 h post infection [21]. Recent published data from our group indicated that this active suppression of proinflammatory responses induced by *B*. *abortus* in trophoblastic cells requires a functional T4SS and the BtpB TIR-containing protein [27]. Increased expression of TOLLIP maybe a host mechanism for controlling inflammation or it may be induced by the pathogen in order to evade an effective immune response. It is known that *Brucella* spp. is able to subvert immune response by the producing BtpA and BtpB that bind directly to MyD88 thereby preventing TLR signaling [10–12].

Another protein that participates in TLR signaling, and that was identified in infected trophoblastic cells in this study was the high-mobility group box 1 (HMGB1), a DAMP (damage associated molecular pattern), which participates in signaling danger to other cells, activates innate and adaptive immune responses and promotes tissue regeneration [28]. HMGB1 is an endogenous ligand of TLR, which may explain its ability to induce cellular activation and inflammatory responses similar to those initiated by LPS [29]. Considering that HMGB1 is passively released by necrotic cells or actively secreted by activated cells of the immune system, its ligation to several receptors that induce inflammation including TLR2, TLR4, TLR9, and RAGE, activating NF-kB and inducing secretion of proinflammatory cytokines, expression of HMGB1 by cell of CAM explants infected with *B*. *abortus* can have a significant immunomodulatory effect and can potentially impact the outcome of pregnancy, and along with other mechanisms may play a relevant role in the development of necrotizing placentitis that is associated with *B*. *abortus*-induced abortion in cattle.

The Ras-related protein Rab-11A (RAB11A) was upregulated in explants infected with *B*. *abortus*. This protein is related to transport of TLR to phagocytic vesicles containing Gram-negative bacteria and consequently with mechanisms of pathogen recognition and immune response [30–32]. Another protein related to intracellular trafficking that was identified in infected CAM explants was SCAMP2 (Secretory carrier membrane proteins 2), which is part of a group of transmembrane proteins expressed in most eukaryotic cells that participate in traffic vesicles between Golgi apparatus and plasma membrane and also plays a role in exocytosis and endocytosis [33]. The involvement of SCAMPs in formation of *Salmonella* sp. containing vacuole (SCV) has been described [34]. Several intracellular pathogens are able to manipulate the secretory pathways of the host cell, including *Brucella* spp. which interacts with the endoplasmic reticulum to establish a niche for multiplication and to evade the host immune response.

The proteomic screening in this study also identified proteins related to generation of reactive oxygen species (ROS), and inflammatory responses induced by oxidative stress (Aldose reductase—AKR1B1, NADH dehydrogenase [ubiquinone] iron-sulfur protein 8—NDUFS8) [35]. Induction of ROS causes loss of intracellular redox homeostasis, with altered cell signaling and development of pathological processes [36]. Moreover, *Brucella* spp. is capable to produce superoxide dismutase (SOD), which protects against endogenous superoxide produced by aerobic metabolism and respiratory burst from the host cell. Production of SOD prevents bacterial death and enables the establishment and maintenance of intracellular bacteria, and thus it is important for the survival of *Brucella* spp. in its intracellular niche [37]. Conversely, biliverdin reductase (BVR), thioredoxin-dependent peroxide reductase (PRDX3—peroxiredoxin 3) and heat shock protein beta-1 (HSPB1) or Hsp27 are proteins related to cytoprotection against oxidative stress and were also identified in *B*. *abortus*-infected CAM explants [38]. Detection of these proteins in infected explants suggests an attempt of host cells to reduce the damaging effects of ROS, although these proteins may also favor intracellular survival of *Brucella* spp.

*B*. *abortus* is capable of inducing a strong neutrophilic and necrotizing placentitis that is associated with transcription of CXCL8 and CXCL6 in CAM explants and *in vivo* in the placenta of experimentally infected cows [21]. In present proteomic analysis of infected CAM explants resulted in the identification of several proteins potentially related to recognition of pathogens and tissue inflammation that had not been reported in previous studies. Among these proteins, there was upregulation of the complement subcomponent1 Q-binding protein (C1QBP), an intracellular protein found predominantly in association with mitochondria and the nucleus whose expression can be activated by the action of proinflammatory cytokines such as IFN-γ, TN F-α, or LPS [39]; protein disulfide isomerase A3 (PDIA3), a protein that belongs to the superfamily of enzymes thioloxiredutases that is specifically associated with peptide presentation via class I MHC and whose overexpression has been recently related to immunological processes [40]; aldose 1-epimerase or galactosemutarotase (GALM), an enzyme that catalyzes interconversion of β-D-galactose to α-D-galactose, whose increase in expression has been described in inflammatory processes [41].

Interestingly, in *B*. *abortus* CAM explants there was low abundance of adenosylhomocysteinase (AHCY), a highly conserved enzyme that catalyzes the reversible hydrolysis of S-adenosyl-L-homocysteine to adenosine and homocysteine so it is considered a key enzyme in immune response. The use of AHCY inhibitors for therapeutic purposes in inflammatory and immune diseases is being studied, since inhibition of AHCY is associated with immunosuppression [42].

## Conclusions

This study clearly demonstrated changes in the protein expression profile of bovine trophoblastic cells in CAM explants in early stages of *B*. *abortus* infection and that infection induces increase or production of proteins that may be associated with the necrotic placentitis seen in infection of cattle placenta. Several of the proteins that were upregulated during infection are associated with modulation of the innate host immune response to infection with *Brucella abortus*. Therefore, this study contributes to improving our understanding of the mechanisms related to abortion caused by infection with *B*. *abortus* in cattle.

## Supporting Information

S1 Fig 2DE gel obtained from CAM explants infected with *B*. *abortus* 2308 showing the expression profile of proteins. The spots with significant differences in expression (P ≤ 0.05) appear circled in green. Spot 1—Spot overexpressed time of 2 h relative to time of 4 h (t = 4.64471, P ≤ 0.05); Spots 2, 3 and 4—Spots overexpressed in time of 4 h post infection versus time of 8 h (t = 6,66817; t = 4,9594; t = 4,71528; P≤ 0,05). L1, L2 and L3—Landmarks: spots used as a reference for comparison between gels.
(TIF)

S1 Table Number of spots present in more than one experimental group and percentage of corresponding spots (Match) between the triplicate gels of each time point in relation to the Master gel (gel with the highest number of spots) and infected control group. (DOCX)

S2 Table Identification of proteins differentially expressed by trophoblastic cells uninfected and infected by *Brucella abortus* 2308 at the times points of 0.5 h and 4 h. (DOCX)

S3 Table Proteins identified in each spot and their predicted and experimental molecular mass and isoelectric point. (DOCX)
