# Supplementary figures and images for: Proteomic Profile of Brucella abortus-Infected Bovine Chorioallantoic Membrane Explants
Source: PLoS One. 2016 Apr 22;11(4):e0154209. doi: 10.1371/journal.pone.0154209 (PMC4841507; doi:10.1371/journal.pone.0154209)

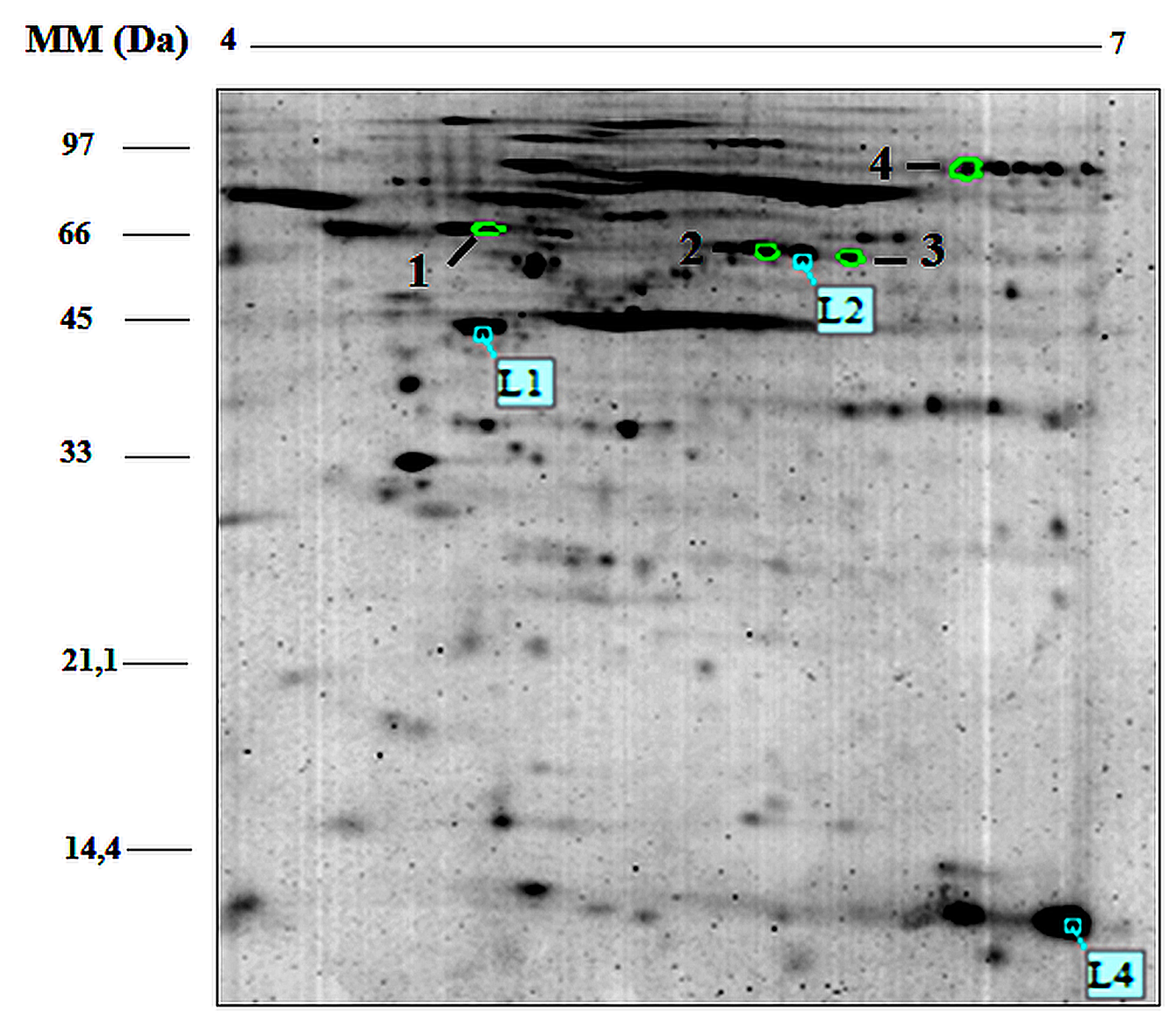

Supplement: S1 Fig — The spots with significant differences in expression (P ≤ 0.05) appear circled in green. Spot 1—Spot overexpressed time of 2 h relative to time of 4 h (t = 4.64471, P ≤ 0.05); Spots 2, 3 and 4—Spots overexpressed in time of 4 h post infection versus time of 8 h (t = 6,66817; t = 4,9594; t = 4,71528; P≤ 0,05). L1, L2 and L3—Landmarks: spots used as a reference for comparison between gels. (TIF) [file pone.0154209.s001.tif]
