# Supplementary material for: Proteomic Profile of Brucella abortus-Infected Bovine Chorioallantoic Membrane Explants
Source: PLoS One. 2016 Apr 22;11(4):e0154209. doi: 10.1371/journal.pone.0154209 (PMC4841507; doi:10.1371/journal.pone.0154209)
Supplement: S1 Table — (DOCX) [file pone.0154209.s002.docx]

Supplementary Table 1 - Number of spots present in more than one experimental group and percentage of corresponding spots (Match) between the triplicate gels of each time in relation to the Master gel (gel with the highest number of spots) and infected control group

| **Experimental Group** | **Master Gel** | **Time points** | **Number of Match** | **% Match** |
| --- | --- | --- | --- | --- |
|  |  | 0.5 h | 78 | 84,3243 |
| Uninfected | 4 h | 2 h | 74 | 77,4869 |
|  |  | 8 h | 73 | 79,3478 |
|  |  | 2 h | 150 | 82,4176 |
| Infected | 0.5 h | 4 h | 160 | 83,7686 |
|  |  | 8 h | 176 | 87,2180 |
