# Supplementary material for: Proteomic Profile of Brucella abortus-Infected Bovine Chorioallantoic Membrane Explants
Source: PLoS One. 2016 Apr 22;11(4):e0154209. doi: 10.1371/journal.pone.0154209 (PMC4841507; doi:10.1371/journal.pone.0154209)
Supplement: S2 Table — (DOCX) [file pone.0154209.s003.docx]

Supplementary Table 2 - Identification of proteins differentially expressed by trophoblastic cells uninfected and infected by *Brucella abortus* 2308 at the times of 0.5 h and 4 h. Spot numbers refers the identification used in Figure 5.

| **Spot number** | **Protein Identification** | **GI** | **Sequence** | **Score** | **% Cover** |
| --- | --- | --- | --- | --- | --- |
| 1 | Transitional endoplasmic reticulum ATPase [*Bos taurus*] | gi\|77735541 | 192 – 210 R.EDEEESLNEVGYDDIGGCR.K + Carbamidomethyl (C)  466 – 487 R.ETVVEVPQVTWEDIGGLEDVKR.E  616 – 638 K.NVFIIGATNRPDIIDPAILRPGR.L | 237 | 10 |
| 2 | Transitional endoplasmic reticulum ATPase [*Bos taurus*] | gi\|77735541 | 192 – 210 R.EDEEESLNEVGYDDIGGCR.K + Carbamidomethyl (C)  466 – 487 R.ETVVEVPQVTWEDIGGLEDVKR.E  616 – 638 K.NVFIIGATNRPDIIDPAILRPGR.L | 237 | 10 |
| 3 | Gelsolin isoform b*[Bos taurus]* | gi\|77736201 | 347 – 368R.DPDQTDGPGLSYLSSHIANVER.V  369 – 393R.VPFDAATLHTSTAMAAQHGMDDDGR.G  407 – 430K.VPVDPATYGQFYGGDSYIILYNYR.H  576 – 597 R.AQPVQVAEGSEPDSFWEALGGK.A | 365 | 12 |
| 4 | Unidentified |  |  |  |  |
| 5 | Gelsolin isoform b [*Bos taurus*] | gi\|77736201 | 347 – 368R.DPDQTDGPGLSYLSSHIANVER.V  369 – 393R.VPFDAATLHTSTAMAAQHGMDDDGR.G  407 – 430K.VPVDPATYGQFYGGDSYIILYNYR.H  576 – 597 R.AQPVQVAEGSEPDSFWEALGGK.A | 365 | 12 |
| 6 | Alpha-fetoprotein precursor [*Bos taurus*] | gi\|77735479 | 82 – 100K.QPAGCLENQVSAFLEEICR.E 2 Carbamidomethyl (C); Gln->pyro-Glu (N-term Q)  305 – 333K.LPTTLELGHCIIHAENDDKPEGLSPNVNR.F Carbamidomethyl (C)  470 – 488 K.QLACGEGVADLIIGHLCIR.H 2 Carbamidomethyl (C); Gln->pyro-Glu (N-term Q)  489 – 509R.HEENPINPGVDQCCTSSYSNR.R 2 Carbamidomethyl (C)  510 – 531R.RPCFSSLVVDETYVPPPFSDDK.F Carbamidomethyl (C)  510 – 536 R.RPCFSSLVVDETYVPPPFSDDK.F Carbamidomethyl (C) | 503 | 18 |
| 7 | Alpha-fetoprotein precursor [*Bos taurus*] | gi\|77735479 | 82 – 100K.QPAGCLENQVSAFLEEICR.E 2 Carbamidomethyl (C); Gln->pyro-Glu (N-term Q)  305 – 333K.LPTTLELGHCIIHAENDDKPEGLSPNVNR.F Carbamidomethyl (C)  470 – 488 K.QLACGEGVADLIIGHLCIR.H 2 Carbamidomethyl (C); Gln->pyro-Glu (N-term Q)  489 – 509R.HEENPINPGVDQCCTSSYSNR.R 2 Carbamidomethyl (C)  510 – 531R.RPCFSSLVVDETYVPPPFSDDK.F Carbamidomethyl (C)  510 – 536 R.RPCFSSLVVDETYVPPPFSDDK.F Carbamidomethyl (C) | 503 | 18 |
| 8 | ALB protein [*Bos taurus*] | gi\|154425704 | 52 – 65 F.SQYLQQCPFDEHVK.L Carbamidomethyl (C)  89 – 100 K.SLHTLFGDELCK.V Carbamidomethyl (C) | 1041 | 22 |
|  |  |  | 267 – 280K.ECCHGDLLECADDR.A 3 Carbamidomethyl (C)  347 – 359K.DAFLGSFLYEYSR.R  360 – 371R.RHPEYAVSVLLR.L  421 – 433K.LGEYGFQNELIVR.Y  437 – 451R.KVPQVSTPTLVEVSR.S  469 – 482R.MPCAEDYLSLILNR.L Carbamidomethyl (C)  508 – 523R.RPCFSALTPDETYVPK.A Carbamidomethyl (C)  529 – 544 K.LFTFHADICTLPDTEK.Q Carbamidomethyl (C) |  |  |
| 9 | ALB protein [*Bos taurus*] | gi\|154425704 | 52 – 65 F.SQYLQQCPFDEHVK.L Carbamidomethyl (C)  89 – 100 K.SLHTLFGDELCK.V Carbamidomethyl (C)  267 – 280K.ECCHGDLLECADDR.A 3 Carbamidomethyl (C)  347 – 359K.DAFLGSFLYEYSR.R  360 – 371R.RHPEYAVSVLLR.L  421 – 433K.LGEYGFQNELIVR.Y  437 – 451R.KVPQVSTPTLVEVSR.S  469 – 482R.MPCAEDYLSLILNR.L Carbamidomethyl (C)  508 – 523R.RPCFSALTPDETYVPK.A Carbamidomethyl (C)  529 – 544 K.LFTFHADICTLPDTEK.Q Carbamidomethyl (C) | 1041 | 22 |
| 10 | ALB protein [*Bos taurus*] | gi\|154425704 | 52 – 65 F.SQYLQQCPFDEHVK.L Carbamidomethyl (C)  89 – 100 K.SLHTLFGDELCK.V Carbamidomethyl (C)  267 – 280K.ECCHGDLLECADDR.A 3 Carbamidomethyl (C)  347 – 359K.DAFLGSFLYEYSR.R  360 – 371R.RHPEYAVSVLLR.L  421 – 433K.LGEYGFQNELIVR.Y  437 – 451R.KVPQVSTPTLVEVSR.S  469 – 482R.MPCAEDYLSLILNR.L Carbamidomethyl (C)  508 – 523R.RPCFSALTPDETYVPK.A Carbamidomethyl (C)  529 – 544 K.LFTFHADICTLPDTEK.Q Carbamidomethyl (C) | 1041 | 22 |
| 11 | ALB protein [*Bos taurus*] | gi\|154425704 | 52 – 65 F.SQYLQQCPFDEHVK.L Carbamidomethyl (C)  89 – 100 K.SLHTLFGDELCK.V Carbamidomethyl (C)  267 – 280K.ECCHGDLLECADDR.A 3 Carbamidomethyl (C)  347 – 359K.DAFLGSFLYEYSR.R  360 – 371R.RHPEYAVSVLLR.L  421 – 433K.LGEYGFQNELIVR.Y  437 – 451R.KVPQVSTPTLVEVSR.S  469 – 482R.MPCAEDYLSLILNR.L Carbamidomethyl (C)  508 – 523R.RPCFSALTPDETYVPK.A Carbamidomethyl (C)  529 – 544 K.LFTFHADICTLPDTEK.Q Carbamidomethyl (C) | 1041 | 22 |
| 12 | Alpha-fetoprotein precursor [*Bos taurus*] | gi\|77735479 | 162 – 168 R.YIYEIAR.R  272 – 283K.GNVLECLQDGER.V Carbamidomethyl (C)  355 – 361R.FTYEYSR.R  551 – 559 K.QQFLINLVK.Q | 252 | 5 |
| 13 | Inositol-3-phosphate synthase 1 [*Bos taurus*] | gi\|114051253 | 176 – 191 R.PSVYIPEFIAANQSAR.A  234 – 250 R.FCEVIPGLNDTAENLLR.T + Carbamidomethyl (C)  466 – 481 K.APLAPPGSPVVNALFR.Q | 347 | 8 |
| 14 | Cytokeratin 8 (370 AA) [*Bos taurus*] | gi\|481 | 133 – 151 R.EMQSQISDTSVVLSMDNNR.N  314 – 342 K.TTSGYAGGLTSSYGTPGFNYSLSPGSFSR.T | 388 | 12 |
| 15 | Cytokeratin 8 (370 AA) [*Bos taurus*] | gi\|481 | 133 – 151 R.EMQSQISDTSVVLSMDNNR.N | 388 | 12 |
| 16 | Cytokeratin 8 (370 AA) [*Bos taurus*] | gi\|481 | 133 – 151 R.EMQSQISDTSVVLSMDNNR.N  314 – 342 K.TTSGYAGGLTSSYGTPGFNYSLSPGSFSR.T | 388 | 12 |
| 17 | Cytokeratin 8 (370 AA) [*Bos taurus*] | gi\|481 | 133 – 151 R.EMQSQISDTSVVLSMDNNR.N  314 – 342 K.TTSGYAGGLTSSYGTPGFNYSLSPGSFSR.T | 388 | 12 |
| 18 | Creatine kinase B-type [*Bos taurus*] | gi\|62751863 | 157 – 172 K.LAVEALSSLDGDLAGR.Y  224 – 236 K.TFLVWINEEDHLR.V  321 – 341 R.GTGGVDTAAVGGVFDVSNADR.L | 382 | 13 |
|  | Cathepsin D [*Bos taurus*] | gi\|262073106 | 48 – 57 K.LLDIACWTHR.K Carbamidomethyl (C)  129 – 139 K.FDGILGMAYPR.I  161 – 169 K.NVFSFFLNR.D  327 – 333 R.YYTVFDR.D | 156 | 9 |
|  | Beta actin [*Bos taurus*] | gi\|194676388 | 86 – 96 K.IWHHTFYNELR.V  240 – 255 R.SYELPDGQVITIGNER.F | 248 | 11 |
| 19 | Serine (or cysteine) proteinase inhibitor, clade B (ovalbumin), member 5 [*Bos taurus*] | gi\|147899296 | 48 – 64 K.GDTADEIGQVLHFENVK.D  65 – 79 K.DVPFGFQTVTSDVNK.L | 187 | 12 |
|  | Activator of 90 kDa heat shock protein ATPase homolog 1 [*Bos taurus*] | gi\|77736277 | 17 – 30 R.ADATNVNNWHWTER.D  213 – 224 R.ESFLTSPEELYR.V | 80 | 7 |
| 20 | Prostaglandin reductase 2 [*Bos taurus*] | gi\|115497482 | 25 – 42R.VEEVNLPDCVNEGQVQVR.T Carbamidomethyl (C) | 128 | 5 |
| 21 | 3'(2'),5'-bisphosphate nucleotidase 1 [*Bos taurus*] | gi\|77736025 | 145 – 167 K.AIAGVINQPYYNYQAGPDAVLGR.T | 169 | 7 |
| 22 | 3'(2'),5'-bisphosphate nucleotidase 1 [*Bos taurus*] | gi\|77736025 | 145 – 167 K.AIAGVINQPYYNYQAGPDAVLGR.T | 169 | 7 |
| 23 | Unidentified |  |  |  |  |
| 24 | Unidentified |  |  |  |  |
| 25 | F-actin-capping protein subunit beta [*Bos taurus*] | gi\|28603770 | 95 – 108R.KLEVEANNAFDQYR.D | 50 | 4 |
| 26 | 3-hydroxyisobutyrate dehydrogenase, mitochondrial precursor [*Bos taurus*] | gi\|114052937 | 61 – 76 K.HGYPLIIYDVFPDACK.E + Carbamidomethyl (C)  150 – 167 K.MGAVFMDAPVSGGVGAAR.S  298 – 311 K.SPILLGSQAHQIYR.M | 388 | 14 |
| 27 | Unidentified |  |  |  |  |
| 28 | Unidentified |  |  |  |  |
| 29 | Transgelin-2 [*Bos taurus*] | gi\|61888874 | 21 – 39K.QYDADLEQILIQWITTQCR.K + Carbamidomethyl (C)  103 – 120R.YGINTTDIFQTVDLWEGK.N | 224 | 18 |
|  | Hemoglobin subunit beta [*Bos taurus*] | gi\|62460494 | 30 – 39 R.LLVVYPWTQR.F  104 – 115R.LLGNVLVVVLAR.R | 103 | 15 |
| 30 | Transthyretin precursor [*Bos taurus*] | gi\|27806789 | 101 – 123 K.SLGISPFHEFAEVVFTANDSGPR.H | 107 | 15 |
| 31 | Dynein light chain roadblock-type 1 **[***Bos taurus***]** | gi\|77735831 | 16 – 31 K.GVQGIIVVNTEGIPIK.S  59 – 70 R.EIDPQNDLTFLR.I + Glu->pyro-Glu (N-term E) | 171 | 29 |
| 40 | Unidentified |  |  |  |  |
| 41 | Unidentified |  |  |  |  |
| 42 | Unidentified |  |  |  |  |
| 43 | LDLR chaperone MESD [*Bos taurus*] | gi\|77735889 | 84 – 104K.RPSAPIDFSQIDPGKPESILK.M  126 – 147 K.ETEEITSLWQGSLFNANYDVQR.F | 199 | 18 |
| 44 | Unidentified |  |  |  |  |
| 45 | Similar to BolA-like protein 2 isoform 1 [*Bos taurus*] | gi\|76653515 | 55 – 73 R.LVNTCLAEELLHIHAFEQK.T Carbamidomethyl (C) | 115 | 22 |
| 46 | Unidentified |  |  |  |  |
| 47 | Unidentified |  |  |  |  |
| 51 | Alpha-actinin-4 [*Bos taurus*] | gi\|148238040 | 167 – 175 K.EGLLLWCQR.K + Carbamidomethyl (C)  301 – 310 R.LASDLLEWIR.R  734 – 745 R.VGWEQLLTTIAR.T  761 – 771 K.GISQEQMQEFR.A | 189 | 5 |
|  | Endoplasmin precursor [*Bos taurus*] | gi\|27807263 | 76 – 84K.FAFQAEVNR.M  385 – 395 K.SILFVPTSAPR.G  396 – 404 R.GLFDEYGSK.K | 145 | 3 |
| 52 | Unidentified |  |  |  |  |
| 53 | Alpha-fetoprotein precursor [*Bos taurus*] | gi\|77735479 | 82 – 100K.QPAGCLENQVSAFLEEICR.E 2 Carbamidomethyl (C); Gln->pyro-Glu (N-term Q)  305 – 333K.LPTTLELGHCIIHAENDDKPEGLSPNVNR.F Carbamidomethyl (C)  470 – 488 K.QLACGEGVADLIIGHLCIR.H 2 Carbamidomethyl (C); Gln->pyro-Glu (N-term Q)  489 – 509R.HEENPINPGVDQCCTSSYSNR.R 2 Carbamidomethyl (C)  510 – 531R.RPCFSSLVVDETYVPPPFSDDK.F Carbamidomethyl (C)  510 – 536 R.RPCFSSLVVDETYVPPPFSDDK.F Carbamidomethyl (C) | 503 | 18 |
|  | Gelsolin isoform a [*Bos taurus*] | gi\|164452943 | 52 – 60M.VVEHPEFLK.A  61 – 71K.AGKEPGLQIWR.V  64 – 71K.EPGLQIWR.V Glu->pyro-Glu (N-term E)  177 – 187 K.HVVPNEVVVQR.L | 173 | 3 |
| 54 | Alpha-fetoprotein precursor [*Bos taurus*] | gi\|77735479 | 82 – 100K.QPAGCLENQVSAFLEEICR.E 2 Carbamidomethyl (C); Gln->pyro-Glu (N-term Q)  305 – 333K.LPTTLELGHCIIHAENDDKPEGLSPNVNR.F Carbamidomethyl (C)  470 – 488 K.QLACGEGVADLIIGHLCIR.H 2 Carbamidomethyl (C); Gln->pyro-Glu (N-term Q)  489 – 509R.HEENPINPGVDQCCTSSYSNR.R 2 Carbamidomethyl (C)  510 – 531R.RPCFSSLVVDETYVPPPFSDDK.F Carbamidomethyl (C)  510 – 536 R.RPCFSSLVVDETYVPPPFSDDK.F Carbamidomethyl (C) | 503 | 18 |
|  | Gelsolin isoform a [*Bos taurus*] | gi\|164452943 | 52 – 60M.VVEHPEFLK.A  61 – 71K.AGKEPGLQIWR.V  64 – 71K.EPGLQIWR.V Glu->pyro-Glu (N-term E)  177 – 187 K.HVVPNEVVVQR.L | 173 | 3 |
| 55 | Alpha-fetoprotein precursor [*Bos taurus*] | gi\|77735479 | 82 – 100K.QPAGCLENQVSAFLEEICR.E 2 Carbamidomethyl (C); Gln->pyro-Glu (N-term Q)  305 – 333K.LPTTLELGHCIIHAENDDKPEGLSPNVNR.F Carbamidomethyl (C)  470 – 488 K.QLACGEGVADLIIGHLCIR.H 2 Carbamidomethyl (C); Gln->pyro-Glu (N-term Q)  489 – 509R.HEENPINPGVDQCCTSSYSNR.R 2 Carbamidomethyl (C)  510 – 531R.RPCFSSLVVDETYVPPPFSDDK.F Carbamidomethyl (C)  510 – 536 R.RPCFSSLVVDETYVPPPFSDDK.F Carbamidomethyl (C) | 503 | 18 |
|  | Gelsolin isoform a [*Bos taurus*] | gi\|164452943 | 52 – 60M.VVEHPEFLK.A  61 – 71K.AGKEPGLQIWR.V  64 – 71K.EPGLQIWR.V Glu->pyro-Glu (N-term E)  177 – 187 K.HVVPNEVVVQR.L | 173 | 3 |
|  | Gelsolin isoform b [*Bos taurus*] | gi\|77736201 | 347 – 368R.DPDQTDGPGLSYLSSHIANVER.V  369 – 393R.VPFDAATLHTSTAMAAQHGMDDDGR.G  407 – 430K.VPVDPATYGQFYGGDSYIILYNYR.H  576 – 597 R.AQPVQVAEGSEPDSFWEALGGK.A | 365 | 12 |
| 56 | ALB protein [*Bos taurus*] | gi\|154425704 | 52 - 65 F.SQYLQQCPFDEHVK.L Carbamidomethyl (C)  89 – 100 K.SLHTLFGDELCK.V Carbamidomethyl (C)  267 – 280K.ECCHGDLLECADDR.A 3 Carbamidomethyl (C)  347 – 359K.DAFLGSFLYEYSR.R | 1041 | 22 |
| 57 | Unidentified |  | 360 – 371R.RHPEYAVSVLLR.L  421 – 433K.LGEYGFQNELIVR.Y  437 – 451R.KVPQVSTPTLVEVSR.S  469 – 482R.MPCAEDYLSLILNR.L Carbamidomethyl (C)  508 – 523R.RPCFSALTPDETYVPK.A Carbamidomethyl (C)  529 – 544 K.LFTFHADICTLPDTEK.Q Carbamidomethyl (C |  |  |
| 60 | Endoplasmin precursor [*Bos taurus*] | gi\|27807263 | 512 – 530 R.FQSSHHPSDMTSLDQYVER.M  640 – 660 R.LTESPCALVASQYGWSGNMER.I + Carbamidomethyl (C) | 178 | 7 |
| 61 | Endoplasmin precursor [*Bos taurus*] | gi\|27807263 | 512 – 530 R.FQSSHHPSDMTSLDQYVER.M  640 – 660 R.LTESPCALVASQYGWSGNMER.I + Carbamidomethyl (C) | 178 | 7 |
| 62 | Heat shock cognate 71 kDa protein [*Bos taurus*] | gi\|76253709 | 37 – 49R.TTPSYVAFTDTER.L  138 – 155K.TVTNAVVTVPAYFNDSQR.Q  221 – 236 K.STAGDTHLGGEDFDNR.M | 197 | 7 |
| 71 | Ribosomal protein P1-like isoform 1 [*Bos taurus*] | gi\|297461273 | 34 – 49 K.AAGVNVEPFWPGLFAK.A | 126 | 14 |
|  | [*Bos taurus*] | gi\|262073073 | 15 – 31Q.EAFSLFDKDGDGTITTK.E  92 – 107 R.VFDKDGNGYISAAELR.H | 283 | 22 |
| 72 | Calmodulin [*Bos taurus*] | gi\|262073073 | 15 – 31Q.EAFSLFDKDGDGTITTK.E  92 – 107 R.VFDKDGNGYISAAELR.H | 283 | 22 |
| 73 | Unidentified |  |  |  |  |
| 74 | Keratin 14-like, partial [*Bos taurus*] | gi\|119938006 | 98 – 115 R.ADLEMQIENLKEELAYLR.K | 226 | 18 |
| 75 | Unidentified |  |  |  |  |
| 76 | Toll-interacting protein [*Bos taurus*] | gi\|90403624 | 147 – 157 R.GPVYIGELPQDFLR.I | 77 | 5 |
| 80 | Secretory carrier-associated membrane protein 2 [*Bos taurus*] | gi\|156120987 | 155 – 164 G.LLQQQEELDR.K | 76 | 3 |
|  | Tropomyosin 4 isoform 2 [*Bos taurus*] | gi\|297466575 | 13 – 27 K.IQVLQQQADDAEER.A  14 – 27 R.KIQVLQQQADDAEER.A  178 – 190 K.YSQKEDKYEEEIK.I | 367 | 11 |
| 81 | Tropomyosin 4 isoform 2 [*Bos taurus*] | gi\|297466575 | 13 – 27 K.IQVLQQQADDAEER.A  14 – 27 R.KIQVLQQQADDAEER.A  178 – 190 K.YSQKEDKYEEEIK.I | 367 | 11 |
| 82 | Biliverdin reductase A [*Bos taurus*] | gi\|147900301 | 29 – 45 R.NPHASSAFLNLIGFVSR.R  148 – 161 K.GSLLFTAAPLEEER.F | 223 | 10 |
| 83 | aldose 1-epimerase [*Bos taurus*] | gi\|77736588 | 7 – 21 R.AVFGDLPLGAGTVEK.F  31 – 46 R.VDIISWGCTITALEVK.D + Carbamidomethyl (C)  52 – 68 R.ASDVVLGFDELEGYLQK.Q | 287 | 14 |
| 84 | Aldose reductase [*Bos taurus*] | gi\|60302887 | 42 – 62R.HIDCAHVYQNENEVGLALQAK.L Carbamidomethyl (C)  178 – 195K.YKPAVNQIECHPYLTQEK.L Carbamidomethyl (C)  204 – 222 K.GIVVTAYSPLGSPDRPWAK.P  270 – 294R.IAENFQVFDFELDKEDMNTLLSYNR.D | 578 | 26 |
|  | Malatedehydrogenase, cytoplasmic [*Bos taurus*] | gi\|77736203 | 221 – 230K.GEFITTVQQR.G  299 – 310 K.VVEGLPINDFSR.E | 139 | 6 |
| 85 | Unidentified |  |  |  |  |
| 86 | Alpha-fetoprotein precursor [*Bos taurus*] | gi\|77735479 | 162 – 168R.YIYEIAR.R  272 – 283 K.GNVLECLQDGER.V Carbamidomethyl (C) | 252 | 5 |
|  |  |  | 355 – 361R.FTYEYSR.R  551 – 559K.QQFLINLVK.Q |  |  |
| 87 | Unidentified |  |  |  |  |
| 88 | High-mobility group box 1-like [*Bos taurus*] | gi\|297483924 | 13 – 24 K.MSSYAFFVQTCR.E + Carbamidomethyl (C)  30 – 43 K.KHPDASVNFSEFSK.K  31 – 43K.HPDASVNFSEFSK.K  113 – 127 K.IKGEHPGLSIGDVAK.K | 266 | 19 |
| 89 | Heat shock protein beta-1 [*Bos taurus*] | gi\|71037405 | 58 – 71 R.ALPAAAIEGPAYNR.A  76 – 90 R.QLSSGVSEIQQTADR.W + Gln->pyro-Glu (N-term Q)  93 – 108 R.VSLDVNHFAPEELTVK.T  168 – 184 K.SATQSAEITIPVTFQAR.A | 517 | 30 |
| 90 | Thioredoxin-dependent peroxide reductase, mitochondrial precursor [*Bos taurus*] | gi\|27806083 | 120 – 149 K.ASEFHDVNCEVVAVSVDSHFSHLAWINTPR.K Carbamidomethyl (C)  219 – 249K.AFQFVEAHGEVCPANWTPESPTIKPHPTASR.E Carbamidomethyl (C) | 253 | 23 |
| 91 | abhydrolase domain-containing protein 14B [*Bos taurus*] | gi\|157428006 | 33 – 42 R.FSVLLLHGIR.F  64 – 74 R.AVAIDLPGLGR.S  117 – 129 Y.SLPFLTAPGSQLR.G  130 – 141 R.GYVPVAPICTDK.I + Carbamidomethyl (C) | 272 | 21 |
| 92 | Unidentified |  |  |  |  |
| 93 | Galectin-7-like [*Bos taurus*] | gi\|119910404 | 58 – 70 R.LDESTVVFNTLER.G  87 – 102 R.GQPFDVLLIATEEGFK.A  103 – 114 K.AVIADSEYHHFR.Y  124 – 137 R.ALEVGGDLQLELVK.I | 430 | 39 |
| 94 | Proteindisulfide-isomerase A3 precursor [*Bos taurus*] | gi\|148230374 | 25 – 38 A.SDVLELTDDNFESR.I  162 – 173 K.DLFSEAHSEFLK.A  259 – 271 K.DLLIAYYDVDYEK.N  352 – 363 R.FLEDYFDGNLKR.Y  434 – 448 K.MDATANDVPSPYEVR.G + Oxidation (M) | 554 | 13 |
| 95 | Beta-hexosaminidase subunit beta preproprotein [*Bos taurus*] | gi\|270483766 | 238 – 252 K.GSYSLSHVYTPNDVR.T  493 – 507 R.LWSPQEVTDLDDAYR.R  520 – 536 R.GIAAQPLFTGYCEHEGR.M + Carbamidomethyl (C) | 292 | 8 |
|  | Keratin, type II cytoskeletal 7 [*Bos taurus*] | gi\|114051856 | 54 – 65 R.SSYGAPVGTGIR.A  152 – 162 K.QLEALQLDGGR.L + Gln->pyro-Glu (N-term Q)  138 – 150 R.LPGIFEAQIAGLR.K  331 – 343 R.SKLEAAIADAEQR.G | 295 | 12 |
| 100 | Placental prolactin related protein 2 precursor [*Bos taurus*] | gi\|333440446 | 165 – 174 K.LQAFIESQFR.K  183 – 193 K.TIHEVSNTWSR.F  194 – 204 R.FSSMTFSDEDR.S | 165 | 13 |
| 101 | Placental prolactin related protein 2 precursor [*Bos taurus*] | gi\|333440446 | 165 – 174 K.LQAFIESQFR.K  183 – 193 K.TIHEVSNTWSR.F  194 – 204 R.FSSMTFSDEDR.S | 165 | 13 |
| 102 | Placental prolactin related protein 2 precursor [*Bos taurus*] | gi\|333440446 | 165 – 174 K.LQAFIESQFR.K  183 – 193 K.TIHEVSNTWSR.F  194 – 204 R.FSSMTFSDEDR.S | 165 | 13 |
| 103 | Complement component 1 Q subcomponent-binding protein, mitochondrial precursor [*Bos taurus*] | gi\|77736001 | 78 – 91K.AFVDFLSDEIKEEK.K  204 – 216 K.EVSFQATGESDWK.D | 177 | 9 |
| 104 | Ras-related protein Rab-11A [*Bos taurus*] | gi\|84000297 | 17 – 26K.STIGVEFATR.S  37 – 47 K.AQIWDTAGQER.Y  50 – 57R.AITSAYYR.G  71 – 79K.HLTYENVER.W | 181 | 17 |
| 105 | Calmodulin [*Bos taurus*] | gi\|262073073 | 15 – 31 Q.EAFSLFDKDGDGTITTK.E  92 – 107 R.VFDKDGNGYISAAELR.H | 283 | 22 |
| 106 | Proactivator polypeptide [*Bos taurus*] | gi\|27806447 | 196 – 214 N.GNVCQDCIQLVTDVQEALR.T + 2 Carbamidomethyl (C)   244 – 263 K.NYINQYSEVAIQMVMHMQPK.E + Oxidation (M) | 208 | 7 |
|  | Prosaposin [*Bos taurus*] | gi\|120419464 | 196 – 214 N.GNVCQDCIQLVTDVQEVLR.T 2 Carbamidomethyl (C)  244 – 263 K.NYINQYSEVAIQMVMHMQPK.E Oxidation (M) | 98 | 7 |
| 107 | ALB protein [Bos taurus] | gi\|154425704 | 52 – 65 F.SQYLQQCPFDEHVK.L Carbamidomethyl (C)  89 – 100 K.SLHTLFGDELCK.V Carbamidomethyl (C)  267 – 280K.ECCHGDLLECADDR.A 3 Carbamidomethyl (C)  347 – 359K.DAFLGSFLYEYSR.R  360 – 371R.RHPEYAVSVLLR.L  421 – 433K.LGEYGFQNELIVR.Y  437 – 451R.KVPQVSTPTLVEVSR.S  469 – 482R.MPCAEDYLSLILNR.L Carbamidomethyl (C)  508 – 523R.RPCFSALTPDETYVPK.A Carbamidomethyl (C)  529 – 544 K.LFTFHADICTLPDTEK.Q Carbamidomethyl (C) | 1041 | 22 |
| 108 | Unidentified |  |  |  |  |
| 109 | Unidentified |  |  |  |  |
| 110 | Unidentified |  |  |  |  |
| 111 | Unidentified |  |  |  |  |
| 112 | NADH dehydrogenase [ubiquinone] iron-sulfurprotein8,  mitochondrial precursor [*Bos taurus*] | gi\|296471574 | 118 – 135K.LCEAVCPAQAITIEAEPR.A 2 Carbamidomethyl (C)  190 – 212 K.LLNNGDKWEAEIAANIQADYLYR.- | 208 | 19 |
| 113 | Unidentified |  |  |  |  |
| 120 | Unidentified |  |  |  |  |
| 121 | Tropomyosin 4 isoform 2 [*Bos taurus*] | gi\|297466575 | 13 – 27 K.IQVLQQQADDAEER.A  14 – 27 R.KIQVLQQQADDAEER.A  178 – 190 K.YSQKEDKYEEEIK.I | 367 | 11 |
| 122 | Unidentified |  |  |  |  |
| 123 | Unidentified |  |  |  |  |
| 124 | Tropomyosin 4 isoform 2 [*Bos taurus*] | gi\|297466575 | 13 – 27 K.IQVLQQQADDAEER.A  14 – 27 R.KIQVLQQQADDAEER.A  178 – 190 K.YSQKEDKYEEEIK.I | 367 | 11 |
| 125 | Unidentified |  |  |  |  |
| 126 | Proteindisulfide-isomerase A3 precursor [*Bos taurus*] | gi\|148230374 | 25 – 38 A.SDVLELTDDNFESR.I  162 – 173 K.DLFSEAHSEFLK.A  259 – 271 K.DLLIAYYDVDYEK.N  352 – 363 R.FLEDYFDGNLKR.Y  434 – 448 K.MDATANDVPSPYEVR.G + Oxidation (M) | 554 | 13 |
| 127 | Beta-hexosaminidase subunit beta preproprotein [*Bos taurus*] | gi\|270483766 | 238 – 252 K.GSYSLSHVYTPNDVR.T  493 – 507 R.LWSPQEVTDLDDAYR.R  520 – 536 R.GIAAQPLFTGYCEHEGR.M + Carbamidomethyl (C) | 292 | 8 |
|  | Keratin, type II cytoskeletal 7 [*Bos taurus*] | gi\|114051856 | 54 – 65 R.SSYGAPVGTGIR.A  152 – 162 K.QLEALQLDGGR.L + Gln->pyro-Glu (N-term Q)  138 – 150 R.LPGIFEAQIAGLR.K  331 - 343 R.SKLEAAIADAEQR.G | 295 | 12 |
| 128 | Ornithineaminotransferase, mitochondrial precursor [*Bos taurus*] | gi\|296472532 | 33 – 46 K.TVQGPPSSDYIFER.E  50 – 64 K.YGAHNYHPLPVALER.G  114 – 129 R.AFYNNVLGEYEEYVTK.L | 269 | 10 |
| **129** | **Adenosylhomocysteinase [*Bos taurus*]** | **gi\|77735583** | **9 – 19 K.VADISLAAWGR.K**  **95 – 103 K.AGIPVYAWK.G**  **143 – 151 K.YPQLLSGIR.G**  **187 – 196 K.SKFDNLYGCR.E + Carbamidomethyl (C)**  **310 – 318 K.WLNENAVEK.V**  **413 – 421 K.QAQYLGVSR.E**  **422 – 431 R.EGPFKPDHYR.Y + Glu->pyro-Glu (N-term E)** | **378** | **17** |
|  | **Adenosylhomocysteinase [*Brucella abortus*str. 2308 A]** | **gi\|237816445** | **79 – 97 R.WSSCNIFSTQDHAAAAIAK.A + Carbamidomethyl** | **61** | **4** |
| 130 | Keratin 14-like, partial [*Bos taurus*] | gi\|119938006 | 127 – 147 R.GQVGGDVNVEMDAAPGVDLSR.I | 226 | 18 |
| 131 | Aldose reductase [*Bos taurus*] | gi\|60302887 | 42 – 62R.HIDCAHVYQNENEVGLALQAK.L Carbamidomethyl (C)  178 – 195K.YKPAVNQIECHPYLTQEK.L Carbamidomethyl (C)  204 – 222 K.GIVVTAYSPLGSPDRPWAK.P  270 – 294R.IAENFQVFDFELDKEDMNTLLSYNR.D | 578 | 26 |
|  |  |  |  |  |  |
|  | Malatedehydrogenase, cytoplasmic [*Bos taurus*] | gi\|77736203 | 221 – 230K.GEFITTVQQR.G  299 – 310 K.VVEGLPINDFSR.E | 139 | 6 |
| 132 | Unidentified |  |  |  |  |
| 133 | Unidentified |  |  |  |  |
| 134 | Heat shock protein beta-1 [*Bos taurus*] | gi\|71037405 | 58 – 71 R.ALPAAAIEGPAYNR.A  76 – 90 R.QLSSGVSEIQQTADR.W + Gln->pyro-Glu (N-term Q)  93 – 108 R.VSLDVNHFAPEELTVK.T  168 – 184 K.SATQSAEITIPVTFQAR.A | 517 | 30 |
| 135 | Thioredoxin-dependent peroxide reductase, mitochondrial precursor [*Bos taurus*] | gi\|27806083 | 120 – 149 K.ASEFHDVNCEVVAVSVDSHFSHLAWINTPR.K Carbamidomethyl (C)  219 – 249K.AFQFVEAHGEVCPANWTPESPTIKPHPTASR.E Carbamidomethyl (C) | 253 | 23 |
| 136 | Abhydrolase domain-containing protein 14B [*Bos taurus*] | gi\|157428006 | 33 – 42 R.FSVLLLHGIR.F  64 – 74 R.AVAIDLPGLGR.S  117 – 129 Y.SLPFLTAPGSQLR.G  130 – 141 R.GYVPVAPICTDK.I + Carbamidomethyl (C) | 272 | 21 |
| 137 | High-mobility group box 1-like [*Bos taurus*] | gi\|297483924 | 13 – 24 K.MSSYAFFVQTCR.E + Carbamidomethyl (C)  30 – 43 K.KHPDASVNFSEFSK.K  31 – 43K.HPDASVNFSEFSK.K  113 – 127 K.IKGEHPGLSIGDVAK.K | 266 | 19 |
