# Supplementary material for: Proteomic Profile of Brucella abortus-Infected Bovine Chorioallantoic Membrane Explants
Source: PLoS One. 2016 Apr 22;11(4):e0154209. doi: 10.1371/journal.pone.0154209 (PMC4841507; doi:10.1371/journal.pone.0154209)
Supplement: S3 Table — (DOCX) [file pone.0154209.s004.docx]

Supplementary Table 3 - Proteins identified in each spot and their predicted and experimental molecular mass and isoelectric point

| *Spot* | **Identificação Proteínas** | ***Mr* pred**  **(Da)** | ***Mr* exp**  **(Da)** | ***pI* pred** | ***pI* exp** |
| --- | --- | --- | --- | --- | --- |
| 1 | Transitional endoplasmic reticulum ATPase | 89233 | 97000 | 5,13 | 5,51 |
| 2 | Transitional endoplasmic reticulum ATPase | 89233 | 96581 | 5,13 | 5,56 |
| 3 | Gelsolin isoform b | 80681 | 97000 | 5,54 | 5,61 |
| 5 | Gelsolin isoform b | 80681 | 93700 | 5,54 | 6,19 |
| 6 | Alpha-fetoprotein precursor | 68543 | 89927 | 5,92 | 6,27 |
| 7 | Alpha-fetoprotein precursor | 68543 | 90317 | 5,92 | 6,16 |
| 8 | ALB protein | 69248 | 85933 | 5,95 | 6,26 |
| 9 | ALB protein | 69248 | 84825 | 5,95 | 6,33 |
| 10 | ALB protein | 69248 | 88767 | 5,95 | 6,38 |
| 11 | ALB protein | 69248 | 87244 | 5,95 | 6,43 |
| 12 | Alpha-fetoprotein precursor | 68543 | 84642 | 5,92 | 6,46 |
| 13 | Inositol-3-phosphate synthase 1 | 60723 | 72434 | 5,68 | 5,88 |
| 14 | Cytokeratin 8 | 42369 | 59392 | 5,13 | 5,90 |
| 15 | Cytokeratin 8 | 42369 | 58985 | 5,13 | 5,94 |
| 16 | Cytokeratin 8 | 42369 | 57384 | 5,13 | 5,91 |
| 17 | Cytokeratin 8 | 42369 | 59120 | 5,13 | 6,02 |
| 18 | Creatine kinase B-type | 42692 | 51638 | 5,47 | 6,23 |
| 18 | Cathepsin D | 44477 | 51638 | 7,56 | 6,23 |
| 18 | Beta actin | 41845 | 51638 | 5,30 | 6,23 |
| 19 | Serine (or cysteine) proteinase inhibitor, clade B (ovalbumin), member 5 | 28167 | 47875 | 5,25 | 5,92 |
| 19 | Activator of 90 kDa heat shock protein ATPase homolog 1 | 38217 | 47875 | 5,43 | 5,92 |
| 20 | Prostaglandin reductase 2 | 38375 | 44677 | 5,34 | 5,89 |
| 21 | 3'(2'),5'-bisphosphate nucleotidase 1 | 33307 | 46468 | 5,34 | 5,99 |
| 22 | 3'(2'),5'-bisphosphate nucleotidase 1 | 33307 | 44870 | 5,34 | 6,00 |
| 25 | F-actin-capping protein subunit beta | 33720 | 34657 | 6,02 | 5,92 |
| 26 | 3-hydroxyisobutyrate dehydrogenase, mitochondrial precursor | 35387 | 34210 | 8,38 | 6,31 |
| 29 | Transgelin-2 | 22412 | 15948 | 8,39 | 6,04 |
| 29 | Hemoglobin subunit beta | 15849 | 15948 | 6,51 | 6,04 |
| 30 | Transthyretin precursor | 15717 | 16969 | 5,91 | 6,47 |
| 31 | Dynein light chain roadblock-type 1 | 10969 | 14815 | 6,58 | 6,54 |
| 43 | LDLR chaperone MESD | 25960 | 19871 | 6,08 | 6,39 |
| 45 | Similar to BolA-like protein 2 isoform 1 | 10094 | 15274 | 5,78 | 6,29 |
| 51 | Alpha-actinin-4 | 104865 | 95369 | 5,27 | 5,81 |
| 51 | Endoplasmin precursor | 92369 | 95369 | 4,76 | 5,81 |
| 53 | Alpha-fetoprotein precursor | 68543 | 94222 | 5,92 | 5,87 |
| 53 | Gelsolin isoform a | 85634 | 94222 | 5,86 | 5,87 |
| 54 | Alpha-fetoprotein precursor | 68543 | 93766 | 5,92 | 5,93 |
| 54 | Gelsolin isoform a | 85634 | 93766 | 5,86 | 5,93 |
| 55 | Alpha-fetoprotein precursor | 68543 | 93540 | 5,92 | 6,00 |
| 55 | Gelsolin isoform a | 85634 | 93540 | 5,86 | 6,00 |
| 55 | Gelsolin isoform b | 80681 | 93540 | 5,54 | 6,00 |
| 56 | ALB protein | 69248 | 93994 | 5,95 | 6,11 |
| 60 | Endoplasmin precursor | 92369 | 96765 | 4,76 | 5,65 |
| 61 | Endoplasmin precursor | 92369 | 97235 | 4,76 | 5,71 |
| 62 | Heat shock cognate 71 kDa protein | 71226 | 94679 | 5,37 | 5,71 |
| 71 | Ribosomal protein P1-like isoform 1 | 11567 | 20332 | 4,26 | 4,06 |
| 71 | Calmodulin | 16836 | 20332 | 4,09 | 4,06 |
| 72 | Calmodulin | 16836 | 18899 | 4,09 | 4,07 |
| 74 | Keratin 14-like, partial | 24618 | 58862 | 4,72 | 4,24 |
| 76 | Toll-interacting protein | 30088 | 34142 | 5,30 | 5,59 |
| 80 | Secretory carrier-associated membrane protein 2 | 43978 | 34391 | 9,03 | 4,81 |
| 80 | Tropomyosin 4 isoform 2 | 28966 | 34391 | 5,02 | 4,81 |
| 81 | Tropomyosin 4 isoform 2 | 28966 | 34895 | 5,02 | 4,67 |
| 82 | Biliverdin reductase A | 33622 | 43711 | 5,85 | 6,69 |
| 84 | Aldose reductase | 36028 | 39829 | 5,88 | 6,91 |
| 84 | Malate dehydrogenase, cytoplasmic | 36415 | 39829 | 6,16 | 6,91 |
| 83 | Aldose 1-epimerase | 37591 | 42644 | 5,89 | 6,77 |
| 86 | Alpha-fetoprotein precursor | 68543 | 36769 | 5,92 | 6,78 |
| 88 | High-mobility group box 1-like | 24921 | 31657 | 5,51 | 6,85 |
| 89 | Heat shock protein beta-1 | 22665 | 28696 | 5,77 | 6,82 |
| 90 | Thioredoxin-dependent peroxide reductase, mitochondrial precursor | 28177 | 27786 | 7,15 | 6,73 |
| 91 | Abhydrolase domain-containing protein 14B | 22441 | 26975 | 6,05 | 6,82 |
| 93 | Galectin-7-like | 15381 | 17261 | 6,08 | 6,80 |
| 94 | Protein disulfide-isomerase A3 precursor | 56894 | 70323 | 6,38 | 6,87 |
| 95 | Beta-hexosaminidase subunit beta preproprotein | 61223 | 66145 | 7,64 | 6,89 |
| 95 | Keratin, type II cytoskeletal 7 | 51546 | 66145 | 5,79 | 6,89 |
| 100 | Placental prolactin related protein 2 precursor | 27665 | 43618 | 7,57 | 4,21 |
| 101 | Placental prolactin related protein 2 precursor | 27665 | 43561 | 7,57 | 4,30 |
| 102 | Placental prolactin related protein 2 precursor | 27665 | 43561 | 7,57 | 4,42 |
| 103 | Complement component 1 Q subcomponent-binding protein, mitochondrial precursor | 30587 | 33810 | 4,75 | 4,31 |
| 104 | Ras-related protein Rab-11A | 24454 | 28043 | 6,12 | 4,06 |
| 105 | Calmodulin | 16836 | 21786 | 4,09 | 4,08 |
| 106 | Proactivator polypeptide | 58013 | 13680 | 5,08 | 4,30 |
| 106 | Prosaposin | 58129 | 13680 | 5,13 | 4,30 |
| 107 | ALB protein | 69248 | 60632 | 5,95 | 6,94 |
| 112 | NADH dehydrogenase [ubiquinone] iron-sulfur protein 8,mitochondrial precursor | 23881 | 26973 | 6,45 | 5,68 |
| 121 | Tropomyosin 4 isoform 2 | 28966 | 33898 | 5,02 | 4,82 |
| 124 | Tropomyosin 4 isoform 2 | 28966 | 40189 | 5,02 | 4,84 |
| 126 | Protein disulfide-isomerase A3 precursor | 56894 | 71176 | 6,38 | 6,87 |
| 127 | Beta-hexosaminidase subunit beta preproprotein | 61223 | 65840 | 7,64 | 6,87 |
| 127 | Keratin, type II cytoskeletal 7 | 51546 | 65840 | 5,79 | 6,87 |
| 128 | Ornithine aminotransferase, mitochondrial precursor | 48088 | 54498 | 6,24 | 6,80 |
| 129 | Adenosylhomocysteinase | 47607 | 55029 | 5,88 | 6,87 |
| 130 | Keratin 14-like, partial | 24618 | 42943 | 4,72 | 6,97 |
| 131 | Aldose reductase | 36028 | 39413 | 5,88 | 6,93 |
| 131 | Malate dehydrogenase, cytoplasmic | 36415 | 39413 | 6,16 | 6,93 |
| 134 | Heat shock protein beta-1 | 22665 | 28880 | 5,77 | 6,83 |
| 135 | Thioredoxin-dependent peroxide reductase, mitochondrial precursor | 28177 | 27778 | 7,15 | 6,73 |
| 136 | Abhydrolase domain-containing protein 14B | 22441 | 27067 | 6,05 | 6,83 |
| 137 | High-mobility group box 1-like | 24921 | 31274 | 5,51 | 6,95 |

Da - Dalton; Mr - molecular mass; pI – isoeletric point; pred – predicted; exp - experimental.
